# On the Treatment of Gun-Shot Wounds

**Published:** 1850-04

**Authors:** 


### REVIEWS.

Art. VI.—*On the Treatment of Gun-Shot Wounds.* By Benj. W. Dudley, M. D., Professor of Surgery in Transylvania University. Transylvania Medical Journal, Vol. 1, No. iii. Dec., 1849.

*On the Use of the Bandage in Gun-Shot Wounds, etc.* By Benjamin Winslow Dudley, M. D., Professor of Anatomy and Surgery in Transylvania University. Transylvania Journal of Medicine, etc., Vol. 1., p. 501. 1828.

It has been remarked that members of the medical profession are prone to pay undue homage to genius, and to bow the knee to authority without that scrutiny which should ever characterise those on whose action often depend the issues of life or death, hence, the thousand instances in which they have been found wandering in a vicious circle, guided by a delusive light, while all nature lay spread out before them, inviting their attention, and offering to all, and yielding to those who accepted the offer, the richest repast of unadulterated truth. Perhaps, no department of surgery has suffered more than the military, by this blind adhesion to authority, and consequent utter neglect of all true sources of information. Occasionally a light shed its rays over the blood-stained battle field, and an error gave place to truth, until the accumulated stores of ages have furnished ample materials to bless the nineteenth century.

The treatment of gun-shot wounds, based upon rigid observation and an impartial analysis of facts, so bountifully supplied by the records of the past, and the experience of the present, is now well up to the high standard of other departments of the science. Rational treatment, marked by simplicity and an adaptation to the indications presented, has supplanted the empyrical and complicated practice of other times, and thousands whose wounds would formerly have resulted in the loss of life or limb, now live to bless the memory of those whose enlightened experience has wrought this change so fraught with good to man.

The time was, when the civil surgeon, who had scarcely ever smelled gunpowder, exhibited a dignified contempt for any and everything that emanated from his brethren, who ministered to those who followed the fortunes of war. The sentiment, thus expressed, was perhaps in some degree justified by the tardy advancement of the science of military surgery. Considering the vast opportunities offered to army surgeons, by the casualties of war, for the most extensive observation of every species of wounds, it is not a little surprising that the science did not keep pace with the art. While many army surgeons attained a nice tact in the dressing and management of desperate wounds, few, during a long period of time, added anything material to recorded science, or succeeded in developing any new or valuable principle of practice. Whether this was owing to the fact that the duties of the military surgeon were usually performed under circumstances highly unfavorable to calm and systematic research, or, that these onerous duties were rendered frequently in the field, where neither time nor convenience permitted the military surgeons to record the particulars and results of their extensive observation, or to other causes, the reproach is not now as just as it was formerly.

Many able surgeons, fresh from fields of blood and carnage in more modern times, have accurately observed and faithfully recorded the valuable information gleaned amid the havoc of war, and the day has gone by, when we may reasonably expect the civil, to instruct the military surgeon in the duties of his peculiar vocation. On the contrary. the former is deeply indebted to the latter, for the overthrow of many fatal errors, that too long stayed the onward march of modern surgery; and for the establishment of numerous conservative principles, the result of diligent inquiry and enlightened experience on many a bloody field, more especially during the continental war that terminated in 1815. To military surgeons, the honor is due of having first contested, and finally, overthrown the pernicious doctrine of Hunter, that amputation must be deferred until suppuration is established, notwithstanding the operation may be clearly and absolutely demanded by the nature and extent of the wound; to them we are indebted for correct information in regard to the practical difference between local and constitutional gangrene; they dissipated the delusion, that it was impossible to command hemorrhage from the large arteries; relieved disarticulation at the hip-joint from the stigma of being a murderous operation, and threw a cheering light on numerous minor points, over which the profession had long groped in darkness.

A moment's reflection will satisfy any one that enlarged observation and experience, which hapily falls only to the lot of surgeons on the field of battle, can alone enable one to appreciate fully, the varied nature and correct treatment of the important class of wounds, which constitutes the subject matter of the essay before us. The philosophical principle, laid down by Percival, is as applicable here as to any other practical branch of medicine. "The materials of just pathology can be drawn only from large masses of observation assembled and arranged in the order of their subjects; nor can durable improvements in practice be established on less full and luminous evidence." Believing this great principle to be true, in all its extent, we feel it due alike to ourself and to our readers to confess our incompetency to do full justice to the subject, upon the consideration of which we are about to enter, if compelled to rely wholly on our own circumscribed observation. Although, in the outset of our professional career, we were thrown where the violence and casualties of frontier life made gun-shot wounds no unfrequent occurrence, and not a few came under our charge, yet we confess that our experience, like that of all civil surgeons, is far two limited to establish a mode of practice, except so far as its correctness and value may have been confirmed by others, who may have been placed amid scenes more favorable to a full and unrestricted study of these complicated accidents. Hence we shall not hesitate to draw liberally on approved authorities in support of many practical remarks which we may have occasion to make on the essay before us.

Our references may appear to be unnecessarily minute and numerous; this, however, will doubtless be pardoned by the reader, when he remembers the influence of the great name that gives weight to the principles contained in this essay, and the remarkably exclusive practice inculcated therein; the utter contempt of its distinguished author, for "the books;" and the high and extraordinary pretension to originality, which forms a distinguishing feature in almost every effusion of his pen, and which is no less prominent in his annual didactic course of instruction. A common introduction to many of the eminent professor's lectures, runs about as follows: "Gentlemen, I regret to say that, in this matter, I stand alone; that I do not know a single author to whom I can refer you for information, who has treated the subject rationally. I would, therefore, impress upon you the vast importance of an undistracted attention to what I am about to say,-embracing, as you will perceive, principles and practice to be learned no where out of this hall."

Another consideration justifies us in the exercise of more than usual care, in bringing to our support the great names, of acknowledged ability, who have won for themselves undying renown, by enlarging the surgeon's sphere of usefulness, and by contributing to the relief of suffering humanity, in numerous cases previously thought to be beyond the reach of the highest professional skill. No one has ever yet been permitted to differ in opinion from Professor Dudley, even on the most insignificant points, without subjecting himself to the serious charge of being actuated by base and unworthy motives. His friends seem to regard him as absolutely immaculate, and they cannot conceive the possibility of an honest difference of opinion between him and others. Hence, no sooner is one of the Professor's doctrines controverted, than the whole kennel rises in fury, and bays forth indignation in a language and style unknown to true science, and which can find a corresponding response only among a certain portion of that debased London population, whose peculiar propensities, habits, manners and diction, are too well known to need illustration.

These unmanly denunciations shall have no influence upon us; on the contrary, it shall be our constant aim to avoid being provoked into the betrayal of an intemperate zeal, which, but too often, usurps the place of knowledge, and shamefully exposes the violence of the unsubdued passions or the intellectual poverty of its unfortunate victims. We would not, willingly, do any injustice to Professor Dudley, and, with every disposition to assign him the rank to which we believe he is fairly entitled, we may be indulged while we briefly scan the eventful history of military surgery, as applicable to the peculiar class of wounds treated of in the essay before us.

When and by whom gunpowder was discovered, is still involved in great obscurity. Roger Bacon was doubtless acquainted with its detonating force, in the twelfth century, but the honor of the discovery is generally awarded to the genius of Berthold Schwartz, a monk of Mayence, who, it is believed, brought it into practical use about the close of the thirteenth or the beginning of the fourteenth century. The Venitians, having acquired a knowledge of the properties of this wonderful compound, availed themselves of it for military purposes about the year 1350.—Immediately afterwards, an acquaintance with its astounding power spread rapidly to every part of Europe, effecting a radical change in the mode of warfare, and introducing to the surgeon a species of wound, marked by decided peculiarities, never before submitted to his care and observation. The general tendency to a belief in the marvellous, which formed so prominent a characteristic of the human mind at that age, very naturally led the uninstructed to attribute the astounding effects of this new and potent agent to the power of magic; whilst the surgeon, speculating on the novel features presented by the wounds inflicted by its agency, regarded them as poisoned, and therefore requiring peculiar treatment; a treatment intended—and, as they supposed, adapted—to eliminate or neutralize the venom, and to obviate the singular effects of the unusual forms of this new missile, and the supposed ustion of the parts. This error became universal, and exerted its cruel influence for ages. Hence the actual cautery, boiling oil, corrosive acids, etc., were everywhere resorted to, inflicting serious injury, and an incredible amount of unnecessary suffering. According to M. Bauden, this practice is, to a certain extent, still in vogue amongst the Arabs.* An ointment highly extolled by Albucasis has, to the present day, maintained its ancient renown, and is held in universal esteem by that notable people.

This error maintained its ascendency and dominated over all minds until the time of that remarkable man, Ambroise Paré, to whom the medical profession, in all its

* Clinique des Plaies D'Armes a Feu. 1836.

departments, is so largely indebted for valuable discoveries and improvements that, in our day, are not unfrequently claimed by men of mediocrity, as the veritable offspring of their own puny intellects. Even Paré was, for a time, misled, and his splendid genius bowed before omnipotent authority, until accident revealed to him the truth, and shed light upon errors rendered venerable and sacred by the lapse of ages.

With his accustomed candor and naiveté, Paré confesses that chance led to the happy discovery. Young, ardent, and unpracticed, he plodded onward in the beaten pathway pointed out by the masters; and his ministrations to the wounded soldiery, who unfortunately fell in the ranks of the army of Piedmont, were in strict accordance with the precepts he had learned. Providence, however, favored a reformation pregnant with good to man. Paré saw, with grief, but a portion of the wounded dressed, when the medicinal agents, at that time believed to be essential in neutralizing the poison of gun-shot wounds, were altogether exhausted. Forced by necessity, he reluctantly resorted to milder dressings, the only kind then at his command, and under the influence of dreadful apprehensions he retired to rest—but not to sleep. Fear agitated his humane heart, and effectually banished sleep from his eyes during the whole of that long and dreary night. With the early dawn he arose to visit the poor disabled soldiers, expecting to find all those dead who had failed to receive the usual treatment. To his great surprize and joy, he found them buoyant in spirits and free from pain and fever, while those whom, the previous evening, he thought most fortunate, were dejected and haggard and writhing in agony, and parching with fever.* All this would have imparted no instruction to a mere routinist, or to one who allows prejudice to subdue rea-

* Œuvers Completes d'Ambroise Paré, tom. ii, p. 127.

son; the one would have continued his silent, thoughtless adhesion to authority; the other, would have discovered naught in it but accident. Not so, however, with Paré; in the beautiful language of one of his own countrymen: "Apotre de la vérité, il la proclama hautement, et se fit des ennemis parmi ces hommes que leur médiocrité a toujours poussés à défendre leurs erreurs et leurs préjugés avec une persévérance désolant."

In spite of the most bitter and unrelenting opposition of ignorance and fanaticism, the great architect of surgery succeeded in dispelling the mischievous and fatal errors, which had wrought so much evil in the management of gun-shot wounds; triumphantly established more correct notions in regard to their nature; and caused a cruel and empyrical mode of treatment to give place to one, the simplicity of which was equalled only by its success.

We cannot but be astonished at the correctness of the views, so far in advance of his age, presented by this wonderful man in a discourse addressed to Charles, the Ninth, on the occasion of the death of the king of Navarre, who was wounded at the siege of Rouen. In this discourse, Paré clearly demonstrated the mode of action, and the peculiar effects of round bodies, and other projectiles on the various sub-systems of the animal economy; and the true causes of the discoloration, and other peculiarities and dangers attending such wounds, are lucidly pointed out.

It is quite unnecessary for us to enter into a full analysis of Paré's doctrines—let it suffice to say, that before his genius the ridiculous theory adopted by Very, Botal, Vigo and others, disappeared as does the morning dew before the rising sun; and with it vanished forever, from civilized surgery, those phantoms of venom and ustion, that had so frightened the surgeon from his propriety, and entailed upon suffering humanity untold evils.

Paré wrote comparatively little upon gun-shot wounds, yet, if we except some complex and antiquated ointments in use at that time, little was added to the invaluable truths he taught, until we approximate closely towards our own age.

Paré's predecessors, as remarked by Richerand, limited themselves to puerile commentaries on the writings of the Arabian authors, and reduced surgery to the use of ointments and plasters;—with rare exceptions, his successors did little better than offer their servile devotions, at the shrine of the master, whom they adored.

About the same period, Maggius, in Italy, nobly met the objections of Sennertus, and lent his powerful energies to the overthrow of the absurd theory that had so long sustained an irrational and barbarous treatment. Not content with ingenious speculations, however plausible, he appealed to observation, and by a rigid course of experiments, instituted for that purpose, demonstrated the non-venomous qualities of gunpowder; and clearly proved that the projectile was never heated to a degree sufficient to burn even the most combustible substances, and, of course, it could have no such effect on the living tissues. These views induced him to adopt a treatment, at once simple, rational, and successful.

The doctrines of these two great masters were sustained with zeal, energy, and uncommon ability, by J. B. Cancano Leone, of Milan, whose influence and unanswerable arguments, drew to their support almost all intelligent surgeons of the day.

The same views were maintained by Fallopius, in France, and Felix Wurtz, in Germany.

Guillemeau and Francis Thanchin taught the important doctrine, that gun-shot wounds differ from others chiefly in the extent of contusion.

William Clowes, at one time a naval surgeon, but subsequently attached to the army, published a work in 1591

on this class of wounds, distinguished especially for an ardent advocacy of mild dressings.

About 1676 Wiseman gave his contemporaries a practical treatise on gun-shot wounds, which was followed two years afterwards by a learned work on the same subject by John Brown.

In the early part of the eighteenth century several German writers published their views, amongst whom stands preëminent the distinguished surgeon Heister, to whose "General System of Surgery and Surgical Cases" we have often referred, with much profit on various important and practical points.

About the middle of this century, John Ranby published his method of treatment, characterised mainly by his advocacy of a free use of the lancet, light, easy dressings, and a sparing use of instruments.

Francis House, a careful and accurate observer, wrote on the same subject in 1742. The French revolution gave occasion for the display of surgical talent which was, not only not neglected, but highly improved by such men as Baron Percy, Larrey, etc., to whom we are indebted for the valuable results of a protracted and enlightened experience.

Hunter's great work was published in 1794, and in spite of some errors, which modern observation and experience have corrected, it yet stands and will ever remain a monument to his extraordinary genius.

The mere enumeration of those who have cultivated this department of surgery since that day, and given the results of their labor to the profession, would occupy more space than can be allotted to this article. The Bells, Chevalier, Richerand, Boyer, Hutchison, Cooper, Guthrie, Hennen, Lebert, Jobert, Blandin, and a thousand others have, in systematic works, or in essays scattered through the annals of the science, labored to shed light upon the nature of gun-shot wounds, and to improve the practice. Of those that have fallen under our notice, we may designate as worthy of special regard, the works of Guthrie, Hennen, Jobert, and Blandin; the two latter had ample opportunity for observation during the bloody scenes of the modern revolutions of Paris, and did not fail to avail themselves of the facilities afforded by well organised hospitals, for a faithful record of their observations. In regard to Hennen's Military Surgery we cordially endorse the complimentary notice of the "London Medical and Surgical Journal," "it is a work of supererogation for us to eulogise Dr. Hennen's Military Surgery, there can be no second opinion on its merits."

It is somewhat remarkable, that neither our struggle for independence, nor our subsequent wars, have contributed much to the improvement of military surgery, at least, we do not remember any original or elaborate work from these sources.

To the numerous authors, already alluded to, we have now to add the name of the distinguished Professor of Transylvania. As his professional career has been passed in civil life, where comparatively few cases of gunshot wounds have afforded him the opportunity to study their nature and treatment, we naturally anticipate that he is induced to write, for the purpose of directing attention to some important matter, which he supposes has escaped the renowned military surgeons, who have cultivated this branch of surgery on an extended field, amid the carnage incident to the conflict of powerful armies. Such will appear to have been his purpose as we proceed. We have taken the liberty to associate with the paper, now before us, an article published by the Professor on the same subject in the "Transylvania Journal of Medicine," etc., 1828, with a view of presenting to our readers, at one glance, all that the Professor has written in regard to this important class of wounds. The present paper opens with the following unqualified declaration:

"Every expression of opinion by authors upon the nature of gun-shot wounds, leaves the impression upon the mind of the reader in behalf of the peculiarity, which involves sloughing of the fibres in the passage of the ball." The same position was assumed by the Professor in 1828, speaking of the liability of gun-shot wounds to run into violent inflammation followed by sloughing, he says: "Hence the surgical axiom that gun-shot wounds must slough, suppurate and heal by granulation. In observance of these views, the propriety of preserving the orifices of the wound free and open is inculcated; while great solicitude is experienced that the whole tract of the ball may be kept open."

This position is not correct in all the extent here given to it. It is true that the authors acknowledge, as a general rule, that gun-shot wounds will suppurate and heal by granulation; yet, many of them confess that they have witnessed the healing of such wounds by the first intention, and inculcate the propriety of endeavoring to effect this desirable end in all favorable cases. S. Bell admits that such wounds do sometimes heal by first intention, but says they "rarely admit of being united by the adhesive inflammation."*

Dorsey confesses that most gun shot wounds "must be allowed to suppurate," but declares that these wounds "do often heal by the first intention."†

Lebert says: "Des trajits des plaies par d' armes à feu guirissent quelquefois en partie ou en totalité par premieré intention."‡

M. Cloquet says, if the wound is not complicated by hemorrhage; if no foreign substance is present, or is removeable with facility, etc., "on peut tenter la réunion

---

* First Lines, 1808, p. 70.

† Surgery, vol. i, p. 52.

‡ Arch. Gen. de Med. Tom. vii, p. 341.

par premiére intention de la base des lambeaux, et leur sommet seul suppure dans ce cas."*

Roche, Sanson, and Lenoir, after alluding to the healing of gun-shot wounds, say: "des plaies résultant du passage de balles de fusil à travers les parties peuvent guérir de la méme maniére. Des faits de ce genre ont été observé par Hunter et par Larrey." They also note the case of a young man in July, 1830, who "avait eu la cuisse traversée de part en part par une balle, et chez lequel les orifices sentiment du trajet ont suppuré, de telle sorte que la guérison a été compléte dans l' espace de quelques jours."†

Bauden says: "La plupart des coups de feu qui n'ont lésé que les parties molles, en ne laissant au milieu d' elles aucun corps étranger, quérissent sans suppuration profonde. Les ouvertures d' entreé et de sortie se ferment par le developpement des bourgeons charnus, et se cicatrisent ensuite comme si la plaie provenait de l' application d' un cautére ou d'un moxa. Les escarres qui tapissent te trajit disparaissent dans ce cas par de voie de la résorption."‡. The same author while combatting an argument in favor of debridement; viz.: that it facilitates the escape of extravasated blood, says: "La force d' asorption sera assez active sans qu' il faille aider à la nature toujours si puissante en parille cas."§

Guthrie says: "An idea is entertained by some, that the whole track of a gun-shot wound must suppurate, and a slough of dead matter be thrown off; but this is an erroneous opinion, taken more as a necessary than a positive fact," etc.||

---

* Dic. de Med. 1827.

† Pathologie Medico-Chirurgicale, Tom. iii, p, 411.

‡ Clinique des Plaies D'armes a Feu., p. 35.

§ Ibid, p. 36.

|| On Gun-Shot Wounds, p. 61.

M. Jobert says: "On a conseillé de réunir, par preniére intention, certaines plaies par armes à feu, celle de la face, par example. Hunter et plusieurs chirurgiens Anglais ont soutenu avec chaleur ce mode de traitement, et ont fait un pompeux éloge de tous ses advantages, parfois méme de ceux qu'il n'a reellement pas. Il est de fait, cependant qu'on a réussi assez souvent en se conduisant comme ils l'ont indiqué, surtout dans les cas où la contusion était moderée. J'ai pet moi-méme, sur les blessés de nos sanglantes journeés de juillet constater la vérité des observations des chirurgiens Anglais," etc.*

The authors generally regard adhesion by the first intention as exceptional, while with Professor Dudley it constitutes the rule. We leave to our readers to determine by observation, who is in the right. Having had the fortune to receive our first instruction from Professor D. we pursued his practice, with implicit faith, until taught by experience, that no single remedy, however valuable, should be relied upon, in the ever varying phases of complicated wounds, to the exclusion of others not less serviceable. We have never failed to resort to the bandage, when the situation of the wound would permit us to avail ourself of the benefits it affords; and we have seen several gun shot wounds closed by adhesive inflammation under its influence; but we must candidly confess, that much the larger number would suppurate, in spite of the vaunted remedy; the former were without serious complication, easily freed from all foreign substances, and in persons of sound constitution. We do not pretend to be as expert in the use of the remedy as Professor D., though we learned the mode of applying it under his instruction, and labored hard to acquire a practical command of its magic power, and we suppose his extraordinary success

---

* Plaies D'Armes a Feu, p. 35.

is fairly attributable to the adroitness with which he adjusts his favorite remedy. We know that when others fail in cases similar to those which he has relieved without difficulty, the never failing reply is—"my friend, the fault is with yourself—you did not apply the roller properly."

The Professor alludes in very proper terms to the influence of the general health on gun-shot wounds. These, like all other injuries, respond to the condition of the system, and heal kindly or take on vicious action, as the constitution may be sound or impaired. Heister's* directions for the general management of the subjects of such wounds are very judicious, and correspond very accurately to the course pursued by Professor D. Suitable caution is also given against injudicious interference; "neither is unwise medication, nor injudicious evacuation less fraught with mischievous consequences in the treatment of gun-shot wounds."

In one particular the Professor's remarks in regard to the constitutional treatment that may become necessary in the management of many cases of gun-shot wounds are exceedingly defective. The whole class of tonics is discarded by the Professor and no substitute is presented calculated to arouse, in time of need, the sinking energies of the system, or to obviate the extreme debility so frequently met with after serious gun-shot injuries. We believe that almost the whole mass of the profession agree on the vast importance of this class of remedies in cases of great exhaustion, as from protracted suppuration, and Professor D. stands almost or quite alone in repudiating every article that belongs to it in such cases, as well as in all others. Tonics are nowhere to be found in all his materia medica; quinine, iron, and everything of

---

* Surgical Cases, pp. 9, 105, etc.

the kind are excluded as inert or hurtful, and nature is left to struggle alone, unarmed, with exhausting disease, and to sink with debility or rally only after weeks of intense suffering and danger, when a few portions of a suitable tonic, seasonably and judiciously administered, would at once turn the scale in favor of life and restore the patient to the enjoyment of health. It is not a little surprising that one who perceives so clearly, and acts so energetically where the system rises above the healthy action, should suddenly stay his hand and leave this same system in exhaustion, without succor, to wrestle with its enemy when depressed as far below, as it was previously above, the healthy point. The demand for sustaining treatment, in certain cases, appears to us to be quite as obvious as the requirements of depletion in others. We cannot deem it necessary to adduce examples in illustration of a matter, about which no one can possibly err who is not so firmly bound to the car of a single opinion, as to be hopelessly beyond the redemption of a change.

In the essay under review, the Professor proceeds to illustrate the fact that engorgements "may be of constitutional or local origin," and remarks:

"But the diversity in the causes productive of engorgement must naturally call forth the exercise of wisdom in the selection of the appropriate remedy."

This is all very well, but the reader is left to "the exercise" of his own "wisdom," as no aid is afforded him "in the selection of the appropriate remedy," nor are any rules given which would indicate the use of one or forbid a resort to another; indeed, there is nothing here but a hint at something very desirable, unaccompanied by the slightest effort on the part of our author to furnish the desideratum. But if the one idea treatment, which our readers will soon perceive pervades the paper before us, be correct, what necessity can there be for "the exercise of wisdom in the selection of the appropriate remedy," or what influence can be exerted in the selection by "the diversity in the causes productive of engorgement?" If a single remedy be appropriate under all circumstances, then all thought and reflection on the subject are utterly useless, and the labor of analysing causes is a wasteful expenditure of time. Where no choice is to be made no investigation can be required.

Mingled with illustrations of the wonderful activity of the absorbent system, (too familiar to the profession to need particular notice), in removing dead as well as living matter, we find the following paragraph, containing the most prominent and important feature of the essay before us:

"Mechanical pressure may be so adjusted as to prevent engorgement, however profound the injury, thereby presenting an absolute and insuperable bar to all mischievous reaction. That fallacious sentiment, so long cherished and ardently urged, wherein the roller is denounced by a large majority, embracing authors of the highest order, cannot resist the power of truth and the triumphs of experience."

In the Professor's former article on gun-shot wounds he asserts, in broad terms: "No surgical author, with whose writings I am acquainted, has suggested the bandage as an appropriate application in gun-shot wounds."

This cannot be regarded otherwise than as an extraordinary confession, since there is scarcely a writer in this department of surgery who does not, in some way, rank bandages amongst the remedial agents appropriate to the treatment of these wounds. Indeed it is scarcely possible to conceive that surgeons who had freely used the bandage in other wounds, prior to the discovery of gunpowder, and who were familiar with its beneficial influence, would at once abandon it in the management of gunshot wounds. True, they did not wholly rely upon it to effect a cure, and we shall hereafter show that it would not then have been, nor is it now, proper to rely on the bandage in all cases. Valuable as we esteem the bandage, we think it will be conceded, by almost all, that there is much to be done in the management of gun-shot wounds besides the proper application of the roller.

If the roller has been ardently denounced by "a large majority, embracing authors of the highest order," we confess we do not know who these authors are. We are fully aware that the abuses of this remedy have been very properly deprecated by many in the warmest terms, and a few may have been driven by these abuses into the extreme of repudiating it altogether; but there is a very material difference between decrying the misuse of a thing and denunciation of the thing itself. We think our readers will be speedily satisfied that the assertion is unfounded, and that Dr. D. does not stand alone in appreciating the value of the roller, save in the excess of his admiration of its miraculous properties.

We may remark that a resort to bandages is regarded by many of the authors so much a matter of course, that they do not allude to them in very direct terms, but seem to take for granted that their value is so well established and so universally conceded that an argument in their favor would be out of place, and that no one will undertake the management of gun-shot injuries without the aid of these truly useful adjuvants.

But let us see if the application of the bandage to gun-shot wounds has not been taught explicitly by others.

Heister says, "the first intention is to use our utmost endeavors to prevent or assuage the tumor and inflammation." This is to be accomplished by dressing "with lint dipped in spirits of wine warmed, covering it with compresses wet with the same liquor." This venerable author wrote elaborately on the use of bandages and says, "they are often medicines of themselves, being the sole application for the cure of the disorder, as in many fractures, luxations, hemorrhages," &c., and "to all the several parts and disorders of the body;" he declares that "recent wounds themselves will very often unite without anything more than dry lint and with a fitting bandage."*

He dressed gun-shot wounds after the example of Magatti and Belloste "but once in twenty-four hours, and many but once in two days, without taking off the whole *bandage*."† He gives particular directions for the application of "longitudinal compresses and bandages in wounds of fire-arms, accompanied by fracture,"‡ &c. The same author relates the case of a soldier who "received a musket ball in his left side, just above the heart, passing out again at the back, between the third and fourth rib, by the scapula." This patient recovered in a few weeks. "The bandage applied the whole time was the napkin about his body, fixed by the scapulary bandage," &c.§

John Bell says: "But it is particularly to be observed that in gun-shot wounds, and other bruised wounds, though it would be imprudent to sew the parts, since it is impossible that they should altogether unite, *yet the gentle and general support which we give by a compress and bandage*, prevents them from separating far from each other, unites the deep parts early, and lessens the extent of that surface which must naturally fall into suppuration."||

Boyer declares, "Le premier parsement doit etre fort simple et tres-doux. On remplit la plaie de charpie mollette qu'on soutient avec des compresses et un bandage qui ne doit pas etre trop serrè."¶

---

*Gen. Sys. Sur. vol. i, p. 53—vol. ii, p. 295, &c.

†Heister's Cases, p. 59.

‡Ibid, p. 62.

§Ibid, p. 76.

||Principles of Surgery, 1812, p. 30. The original was published in 1801.

¶Maladies Chirurgicales, vol. i, p. 508.

Bauden, giving an account of his change of opinion in regard to the supposed necessity for debridement, says: "Les blessures furent panseès simplement; un bandage roulè et contentif constantment arrosé d'eau froide fut appliqué sur le membre dans toute son entendue; * * * la guèrison s'opèrait avec calme et rapiditè."*

Liston believes that suppuration is almost inevitable, but says: "it will be necessary to afford sufficient support to the parts by bandaging."† Nelaton embraces similar views‡

Hennen advised the use of the roller as the most suitable dressing on the field, and this, if found secure, was not removed until after the lapse of several days, depending on the season, climate, and the constitution of the individual or the peculiarity of his wound. He also commends the roller in edema, a frequent consequence of gun-shot injuries, and often complicated by pressure of the lymphatics, or injury to the nerves. In these cases the roller is aided "by gentle friction with moderately stimulating embrocation, succeeded by the local shower bath."§

The same distinguished surgeon declares: "A firm elastic bandage is an indispensable assistant in the cure; the motions of the ribs are not only restrained, but the parts are powerfully supported by its application; if fracture has taken place in any of the bones we have no other means so perfect for retaining them in their place; if a slight degree of emphysema has occurred before we see the patient, we thus prevent its further diffusion, and if we are called on before it takes place we may prevent its occurrence altogether. The extent and tightness of the bandage should be such as to oblige the patient to perform respi-

*Plaies d'Armes à Feu, p. 11.

†Surgery, p. 182.

‡Military Surgery, pp. 48, 71, 168.

§Pathologie Chirurgicale, vol. i., p. 206.

ration as much as possible by the aid of the diaphragm, and of the abdominal muscles."*

The following extract from the same author covers at once, with great force and brevity, almost all the curative properties of the bandage: "In the application of the bandage, however, nicety is essential, as on its due employment the removal of existing evils and the prevention of many more entirely depend. As a support to parts requiring approximation or separation; as preventing the insinuation of matter, blood, or serum among the interstitial spaces; as expelling them and preventing their accumulation when formed; as repressing redundant or protruding growths, or stimulating their absorption, and finally as retaining other applications in contact to the parts; too much attention can scarcely be paid to the application of the roller, and yet candor compels me to say that foreigners of almost all countries excel us in this fundamental part of our art. Our young students may study, philosophise, and reason well; but neither books, reflection, nor arguments will teach the application of a bandage without repeated practice."

The most judicious medical treatment and the ablest surgical operation will fail, if not assisted by good bandaging, and errors in both will soon be recovered, if a proper system is adopted."†

The only difference, apparently, between this deservedly celebrated military surgeon and our author, consists in the exclusiveness of the latter, adopting the bandage as the sole remedy, while the former, gives to it its proper rank without neglecting other agents of equal, and, on many occasions, of superior importance.

A number of cases are detailed in Professor Dudley's

---

*Military Surgery, p. 295.

† Military Surgery, p. 77.

essay with more or less brevity in illustration of the treatment proposed. The first is that of a young man who was accidentally wounded "while he was getting over a fence." "The ball entered midway in the left thigh, and lodged on the outer part of the limb when it was distinguishable to the touch. Its position among the extensor muscles rendered its removal necessary." The dressing consisted of a roller embracing the whole limb from the toes to the hip, with a compress over the track of the ball. The dressings were kept wet with warm spirits during the first four days. "On the tenth day from their application, the dressings were all removed, when the limb presented a healthy appearance, the wound having healed by the first intention."

*Case* 2. "A man received a gun-shot in the upper and back part of the thigh, the ball ranged downwards and passed out on one side of the knee; thus traversing the whole extent of the thigh." This wound was dressed and treated as in the preceding case. "On the tenth day, the first and only dressing in this case was removed, and the patient dismissed well."

The narration of this case is broken by a eulogy on the miraculous powers of the roller, and a brief statement of the Professor's views in relation to "the virtues of cold water dressing," of which nothing was heard "until after the battle of Waterloo." The Professor says: "My own experience is altogether in behalf of warmth united to moisture; the injury resulting from reaction, when it is applied cold, is thereby avoided; and spirit is preferred in all cases of a doubtful character to water, because of the increased evaporation from the surface of the bandage, while subcutaneous absorption is promoted thereby."

A great deal was heard of cold water dressings long before the battle of Waterloo. As remarked by Hennen, the history of the employment of water as a dressing to wounds is curious and instructive. It was a common remedy, and held in high esteem among the Italians, immediately after the discovery of gunpowder, although its virtues were attributed to the mysterious and senseless incantations, at that time, believed to be necessary to impart to the simple fluid healing properties. Blondus published an essay on its efficacy, at Venice, in 1542, which was followed by similar works by Fallopius, Palatius, Joubert and Martell—all published before the beginning of the seventeenth century.

Joubert reprobates the resort to incantation, and prescribes the water: " sans aucun pronouncement de verbis metaphoriques, ne sur icelle, ni sur les drapeaux et charpie." He gives the following opinion of the advantages of the water dressing: " Je pense qu'un des principaux moyers pour haster le geurison des playes est de les tenir bein nettes; or est il que l'eau les nettoye et deterge bein fort. L'eau par sa froideur empesche l'inflammation, tempere l'ardeur des humeurs," etc.*

We may venture to give our own experience, without presumption when we declare that we do not flatter ourself that it is to outweigh that of our venerable author. We have not found either the warm or cold dressing applicable to every case, and we habitually make the preference to depend on the sensation of the patient, nor have we ever failed to derive most advantage from that which imparted the most agreeable sensation to the sufferer. Sometimes one would prove most soothing, while the other inflicted excruciating pain; in a different person, and a similar wound, we have found a complete reversal of these things.

But to return, a case very similar to the one, we have quoted, is reported by the Professor in the first essay already alluded to; the ball entered " the inferior part of the thigh, and came out near to, and on one side of the knee."

---

* Apologie pour les Chirurgiens. 1601.

In this case the dressings were removed on the eigth day; when the wound was found perfectly healed.

*Case* 3.—"The ball entered the integument in the epigastrium, coursed directly downwards, and passed out in the immediate vicinity of the right external abdominal ring; reëntered and opened the scrotum extensively on that side; and lastly inflicted a large but superficial wound on the inner part of the thigh."

A thick compress was applied to the whole extent of the wound, and secured by a properly adjusted bandage. "Upon the removal of the dressings, eight days after they were applied, the wound had healed throughout by the first intention." The Professor adds: "Yet doubtless they were instrumental in the development of a large abcess, immediately adjacent to and in advance of the ileum." It is remarkable when a truly great intellect seises upon a few important principles, whether true or false, how pliantly they are bent to his will, and forced to apply, wherever it is desirable to their master. The Professor calls to his aid the disputed principle, that inefficient compression develops hemorrhagic excitement, but the bearing it has in the connection in which it is placed we do not exactly comprehend. We give our readers the benefit of the whole paragraph:

"Inefficient compression, whether made by the accumulation of blood in the wound, or from the application of dressings, develops hemorrhagic excitement, and causes thereby an alarming loss of blood from vessels which under other circumstances would elicit no attention: and although by the aid of compression, union by the first intention was effected in this case, yet it is doubtful whether deep seated abscesses can always be prevented by the same means, where the wound involves parts that are necessarily in motion, as connected with the great vital processes."

The Professor thinks this cannot be urged as an objection to the use of compression, since "recovery is notwithstanding more speedy, more certain, while the confinement is divested of much suffering through its agency."

*Case* 4. A collegiate pupil was wounded by the accidental discharge of a rifle. "The ball entered a little behind and below the hip; passing deep among the abductors of the thigh, it lodged immediately in the vicinity of the rectum, from whence it was extracted without loss of time." After thoroughly evacuating the bowels, compresses were applied and firmly maintained in place by the T bandage and roller. The bowels were moved by oil on the sixth day; three days afterwards "the entire restoration to health of the whole wound rendered farther dressing unnecessary."

*Case* 5. The ball "passed between the urethra and corpora cavernosa, opening extensively those three bodies; thereby giving a free passage to the contents of the bladder; with blood from the cylindrical bodies. The ball then entered the left scrotum, and left bare the testicle and spermatic chord. In its course it removed a large portion of skin from the front of the left hip; and, lastly, entering the outer part of the wrist, it passed between the skin and extensor tendons of the fingers to the styloid extremity of the ulna, where it made its final exit."

The usual dressings were applied. "At the expiration of a week, all the wounds, with the exception of that on the hips, were closed, and at the removal of the second dressing, twelve days from the injury, he was well."

This case was reported by the Professor in 1828, and the result of the case as there given is: "the wounds of the wrist, cavernous bodies, urethra and scrotum healed by the first intention, *except a slight suppuration from the latter.*"

*Case* 6. A school boy "lost the ends of the fingers of one hand." Dressed with adhesive plaster and the roller. Those fingers which retained sufficient integument to cover the extremity of the stump, healed by the first intention; the others suppurated and healed by granulation, "without anything bearing an approximation to sloughing."

*Case* 7. "The ball (of large caliber) entered the right thigh and opened the femoral artery, causing a gush of arterial blood, which projected several feet from either orifice. Passing, in its course, behind the bone of the left thigh, it inflicted a most distressing wound upon the left femoral nerve." The usual dressings were applied. On the eleventh day no pulsation was discovered in the posterior tibial artery; three days afterwards, the fourteenth from the time of the infliction of the wound, the anterior tibial artery was examined, and gave satisfactory evidence "of healthful arterial circulation."

The dressings were removed on the eighteenth day, "when the very agreeable evidence was set forth, of union by the first intention having been completed, with the exception of a little integument, throughout the wound."

The Professor is evidently not as careful as he should be, in regard to dates, thereby leaving points to which the captious may, hereafter, take exception. In the account of this case, published in 1828, it is stated that pulsation was discovered on the twelfth day, and "on the fourteenth day the entire bandage was removed from the limb," and the wound found as described in the present essay.

These discrepancies may appear to be slight matters, and we feel confident that they may be satisfactorily explained, but the time will come, when they will be pointed to as evidence of want of care and accuracy, and thus throw discredit on the whole case. The discrepancies are nothing to those who know the venerable writer, and appreciate his nice sense of honor and sterling integrity, but posterity will not regard a difference of four days in the statement of the same case, by the same gentleman, as a slight matter; especially, in a case so remarkable as the one before us.

Although the wound in the left thigh healed kindly, yet the patient suffered intensely from irregular muscular action, during several months, and as stated in 1828, he had not " entirely recovered the third year after, when he left this country."

"Similar distressing consequences to wounded nerves," have been under the charge of the Professor very often. Three cases are briefly stated, in illustration of these wounds. A lady, from St. Louis, received such an injury from a lancet, in being bled, from which there resulted " an alarming affection of the arm, head, chest and spine."

In another case, a lacerated finger, amputation was resorted to after much tedious suffering. The operation was not followed by relief, and " it became necessary for the gentleman to use a considerable amount of evacuant medicine, to relieve himself from the effects of morbid secretion." Would not the same treatment have accomplished the end without a resort to amputation?

The third case was that of " a gentlemen who was bitten on the hand and wrist, by a dog." It was only after months that blisters, severe regimen, and evacuants "subdued the pains so far as to rely upon the efforts of nature to restore his health."

The material, of which the gun-cap is composed, is more unfriendly to a wounded surface than a leaden ball, and the Professor declares that wounds inflicted by their explosion, " may be classed among the most critical of those which are of similar extent and locality, the product of fire-arms." Two cases are alluded to, in each of which, a small piece of the cap passed through the cornea and lodged on the anterior surface of the iris. In both cases the wound of the cornea healed by the first intention, but it was indispensable to the preservation of the eye, to remove the foreign substance by an operation similar to that for cataract.

To illustrate Prof. D.'s practice in gun-shot wounds, involving fracture of the bone, the following cases are given.

*Case* 8. A rifle ball "carried away the major part of the magnum, and lodged deep among the muscles of the thigh. After removing isolated fragments of bone, the usual dressings were applied. On the tenth day "the interior wound was occupied by organized matter," and on "the removal of the second dressing, a cicatrix covered each orifice." The wound in the thigh was found healed on the removal of the first dressing.

*Case* 9. "A gentleman received a pistol shot, which shattered the humerus a little above the elbow and separated its condyles from each other." The fractured bone having been properly adjusted, compresses and the roller were put in requisition in the usual manner, "and at the expiration of a month the use of the arm was restored."

The Professor remarks: "the intelligent of the profession are doubtless surprized that, in this case, splints and machinery to control muscular contraction and prevent deformity, should be overlooked." We doubt not our author is correct, for while every surgeon seeks to adjust the bandage to such wounds with as much nicety as possible, prudence would dictate the additional security guaranteed by properly adjusted splints. We tried the practice of relying alone on the bandage, but were finally compelled, under the guidance of experience, to withdrew a portion of the confidence we had been taught to repose in the bandage alone, in such cases.

*Case* 10. A rifle ball, of large size, "entered the scalp on the anterior and lateral portion of the head, of the right side, and fractured the parietal bone. Fragments of bone were removed, and the wound dressed with compress and roller, and healed by the first intention." A few months after recovery he became the subject of epilepsy. A free incision was made through the scalp, "the dura mater, with the interior membranes was opened to the extent of two inches, when, by the aid of forceps, a fragment of bone, one inch long, by half that width, was drawn from deep within the substance of the middle lobe of the cerebrum." The wound healed by the first intention, and there was no return of the epilepsy.

In the first of the Professor's essays he remarks:

"The very common practice of amputating limbs, because of compound fracture by gun-shot wounds, will be suspended when the bandage is properly appreciated. In several cases within my own practice, I have reduced them to the condition of a simple fracture, and have thus managed them with the same facility as in other instances where similar violence has been done in a different way."

Now, we do not believe the practice here spoken of to be common, while we know the bandage was properly appreciated in such cases, and the practice inculcated by the Professor was pursued long before he either adopted or taught it.

In 1707, Heister* met at Brussels several soldiers who had been severely wounded in the bloody action of 1704, near Shellenberg, in Bavaria, and notwithstanding there were compound fractures, and some deeply affecting the joints, every means had been tried to effect a cure, and these efforts were persevered in for a period of three years, before a resort was had to amputation.

The same author gives particular directions for dressing such wounds, not differing materially from the practice pursued by Professor Dudley. "When the wound is first dilated, cleansed, and freed from the splinters of the bone, and other heterogeneous bodies, particularly in

---

*Cases, p. 40.

the upper arm, the shattered bones are again glued together, as in simple fractures, the wound being dressed with lint and vulnerary ointments, and, lastly, with the vulnerary balsam, and a plaster laid over all, but not for both ends to meet, and compresses laid longitudinally round the fractured part, with proper splints and bandage as in common fractures."*

J. Bell gives directions for dressing such wounds without amputation.† He declares that "a limb having the great bones broken, by a musket ball piercing the limb, may be saved." He advises immediate amputation only when the limb is so crushed that an attempt to save it would endanger life, and when, if saved, it would be distorted, and rather an encumbrance than a help.‡

Liston takes a correct view of this matter. He says: "He [the surgeon] must not proceed with his knife when there exists even a slight chance of preserving the limb."§

M. Jobert has succeeded in saving limbs, after amputation had been proposed, under the following circumstances:

1. Penetrating wounds; with fracture of the elbow-joint.

2. Comminuted fracture of the head of the humerus, without destruction of the soft parts.

3. Fractures of the wrist and hand.

4. Comminuted fractures of the thigh, without disorganization of the soft parts.

5. Penetrating wounds of the knee-joint, with or without fracture.

6. Comminuted fracture of the leg, when the soft parts are not too much torn, bruised, or destroyed, notwith-

---

*Cases, p. 62.

†On Wounds, 1807, vol i., p. 158.

‡On Wounds, vol. 2, pp. 174, 175.

§Elements of Surgery, p. 182.

standing there may be several wounds and fragments of bone.*

The Professor declares that he has "long since been convinced of the error propagated in the profession, regarding the peculiarity these gun-shot wounds." In regard to their distinctive character, there does not appear to be much, if any, disagreement between Prof. Dudley and other authors. Since the time of Paré, gun-shot wounds have not been regarded as differing from other wounds, "except in the degree of violence done the wounded surfaces."

Heister declares that they differ from incised wounds in as much as the parts are "more shattered and torn.†"

Hennen's definition is alike comprehensive and accurate: "A gun-shot injury is a violent contusion, with or without solution of continuity, suddenly and rapidly effected by a solid body projected from firearms; and nothing more."‡

The Surgical Lectures of Sir A. Cooper are said to have excited the highest degree of admiration by "the rich tissue of authentic facts and legitimate deductions which spread itself over every page in the work, unsullied by any predominating theory or hypothesis which, like a vapory atmosphere, distorts every object seen through it." We regret that we do not feel authorised to pronounce the same eulogy on the essay before us—the facts are doubtless authentic, and they are either extraordinary or common-place, but they are not sufficiently numerous or varied, nor of a character to serve as a secure basis for an exclusive practice applicable to gun-shot wounds under all circumstances! The bandage theory every where sheds a hazy atmosphere, that will not

*Bulletin de l'Académie, tom. xiv., pp. 64, etc.

†Gen. Sys. Sur. vol. i, p. 51.

‡Military Surgery, p. 48.

permit a clear perception of all the phases presented by this species of wounds, or allow a recognition of the multiplied accidents that do, and must inevitably, occur under any plan of treatment, and which, in the estimation of the first men of the profession, call for something more than the mere support of a bandage.

The unpracticed reader of Professor Dudley's essay will imagine that he has found, in the roller, a sovereign remedy for every variety of gun-shot wounds, from a simple abrasion of the skiu to a compound comminuted fracture of the bone, etc.; but we ask our readers to turn again to the cases reported, and give them a careful examination, when they will be satisfied that they are either selected cases, or that Professor D. has been wonderfully fortunate in meeting only with such as were most favorable to adhesive inflammation. In every case the ball passed out or was readily removed, and with it all foreign substances which might, and probably would, have occasioned suppurative action.

He who goes forth among diseased actions, relying alone on the practical views, inculcated in the paper before us, is doomed to sad disappointment, and cannot but be wofully at a loss for information, not to be found in this "full record" of the Professor's experience. Where is the instruction in regard to those cases where debridment is required? In what manner is the examination to be conducted? What cases require immediate amputation? Should gangrene occur what treatment is demanded? At what point shall the tonic supplant the antiphlogistic treatment? What is to be done in cases of superabundant, deficient, or unhealthy suppuration? For consecutive abscess, marasmus, colliquative diarrhea, etc.? The essay is in fact defective in information in regard to all the consecutive phenomena, accidents and complications which, in spite of the best directed efforts of the surgeon, so frequently supervene in gun-shot wounds.

These injuries are to be treated on rational principles, and there are none that require the exercise of a sounder judgment; the bandage is indeed a valuable remedy; but, if applied to every case, and wholly relied upon in all, it will be converted from a blessing into a curse. Gun-shot wounds present a variety of phases; there are none in which more violence is done, or in which complication is more common, and the experience of the ablest and most indefatigable observers has demonstrated the absolute necessity for a corresponding modification of treatment: at one time, depleting; at another, tonic; at another, soothing; if the wound be irritable, stimulating; if inert, gently escharotic; if the granulations be too exuberant, the appropriate means must be addressed to that condition. Possibly it may be said that we have no right to dictate how far the author shall go in communicating his professional views; certainly we have not, nor should we lay so much stress on the very imperfect account Professor Dudley has given of this class of wounds, but for the purpose of cautioning the young reader that there is much to be learnt in relation to gun-shot injuries, besides what is found in this essay.

In the first announcement of these essays we were told they were to "present a full record" of the Professor's extensive experience, but the subsequent qualification prepared us for a mere abridgement of his views, but certainly did not lead us to anticipate the total omission of many of the most common and important features of the wounds of which he undertakes to tread. It is to be presumed that the Professor has met with cases of gun-shot wounds where foreign substances were retained in the wound, and where accident, complicated and rendered difficult their management, and to complete the record of his experience, some account of these cases and the results of his treatment were necessary. To base a general practice on these few and peculiar cases, would violate one of the soundest principles of medical logic, so clearly stated by Sir G. Blane: "It is only by computation, founded upon large averages, that truth can be ascertained, and hence the danger of founding a general practice on the experience of a single case or a few cases." In the Professor's first essay a few "cases are given by way of contrasting the effects of the ordinary treatment with those of the bandage." We are not informed in what this "ordinary treatment" consisted, but, aside from this, how unfair is the comparison? In these cases the balls, and probably other foreign substances, were retained in the wounds, while in all the cases given to illustrate the superior efficacy of the bandage, the wounds were entirely freed from all such causes of irritation. It is assuredly unfair, and especially averse to the spirit of medical philosophy at this day, to contrast the results of different treatment, unless there be a striking similarity in the cases submitted to the test. It would be quite as fair to select only the cases that have run into suppuration under the influence of the bandage and adduce them to establish the inutility of that valuable remedy. There is not a remedial agent at the command of the profession that might not be banished from use by the same flimsy argument. The truth is, there should be no selection of cases on either side, no contrasts between extremes; let the failures be reported with the more fortunate results, and the profession will be able to arrive at a correct conclusion in regard to the value of different modes of treatment.

In allusion to these unfortunate cases the Professor remarks there can be very little question but that they "were susceptible of cure in a few days by the bandage, "yet he immediately afterwards admits that two of the three cases "were constitutional in the origin of most of their symptoms, after the first effects of the wound subsided. Now how the bandage could have obviated these symptoms, of constitutional origin, we cannot perceive; and highly as we estimate the remedy, in its legitimate sphere, we do not believe that it possesses any such wonderful power. Our author himself admits, in the same arti cle that "partial suppuration supervened in these cases," how then are we reasonably to expect the bandage to effect "a cure in a few days."

There are two errors, the one or the other of which has displayed itself conspicuously in every age of medical science, viz.: first, a disposition to confound many conditions of the system totally and radically different, and thereby to limit the curative resources of the profession to a few simple agents, under all forms of disease. The second error is equally objectionable, as it runs to the opposite extreme, and consists in a species of refinement that engenders shades of complexity in all things, and divides the treatment of disease into an endless variety of means, compared with which the written signs of the Chinese language are simplicity itself. In a practical point of view, our author and some of the ancients occupy the two extremes. We verily believe that the former has applied the bandage, calomel, and tartar, to more purposes, and varied conditions, and probably effected more with these simple agents, than any person who now lives, or who has heretofore recorded his experience; at the same time, we doubt not, he would have accomplished infinitely more, if he had been unshackled by self-imposed restrictions that were unnecessary. The prominent errors alluded to above, naturally lead to the same practical result. While the refined systems were well calculated to introduce inextracable confusion, and to cast so many difficulties in the path-way of truth, that the inquirer, however ardent his temperament, was not unfrequently discouraged and driven from the prosecution of a study which appeared to him to be too complex to be comprehended or useful; the other system, in its simplicity, tends to repress observation, and to limit the laborious effort of searching into the hidden and often almost inappreciable differences of morbid phenomena, that must in the nature of things demand a corresponding modification of treatment.

In medicine and surgery, as in all the practical affairs of life, those who occupy a just medium between the two errors are in the right path—while they investigate as minutely as possible all the varied forms of disease submitted to their observation, they abjure excessive, speculative, and impracticable refinement, and are ever ready to assign to each remedy its appropriate place without the deification of any particular one. C.

